# The Antimicrobial Peptide LJ-hep2 from *Lateolabrax japonicus* Exerting Activities against Multiple Pathogenic Bacteria and Immune Protection In Vivo

**DOI:** 10.3390/md20100651

**Published:** 2022-10-20

**Authors:** Ruihao Gong, Zhe An, Weibin Zhang, Fangyi Chen, Ke-Jian Wang

**Affiliations:** 1 State Key Laboratory of Marine Environmental Science, College of Ocean & Earth Sciences, Xiamen University, Xiamen 361102, China; 2 State-Province Joint Engineering Laboratory of Marine Bioproducts and Technology, College of Ocean & Earth Sciences, Xiamen University, Xiamen 361102, China; 3 Fujian Innovation Research Institute for Marine Biological Antimicrobial Peptide Industrial Technology, College of Ocean & Earth Sciences, Xiamen University, Xiamen 361102, China

**Keywords:** *Lateolabrax japonicus*, hepcidin, antimicrobial peptide, antimicrobial activity, multidrug-resistant bacteria, immunoprotective effect

## Abstract

Hepcidin is widely present in many kinds of fish and is an important innate immune factor. A variety of HAMP2-type hepcidins have strong antimicrobial activity and immunomodulatory functions and are expected to be developed as substitutes for antibiotics. In this study, the antimicrobial activity of Hepc2 from Japanese seabass (*Lateolabrax japonicus*) (designated as LJ-hep2) was investigated using its recombinant precursor protein (rLJ-hep2) expressed in *Pichia pastoris* and a chemically synthesized mature peptide (LJ-hep2_(66–86)_). The results showed that both rLJ-hep2 and synthetic LJ-hep2_(66–86)_ displayed broad antimicrobial spectrum with potent activity against gram-negative and gram-positive bacteria, and fungi. Especially, LJ-hep2_(66–86)_ had stronger antimicrobial activity and exhibited potent activity against several clinically isolated multidrug-resistant bacteria, including *Acinetobacter baumannii*, *Escherichia coli*, *Pseudomonas aeruginosa*, *Klebsiella pneumoniae* and *Enterococcus faecium*. Moreover, LJ-hep2_(66–86)_ exerted rapid bactericidal kinetic (killed tested bacteria within 2 h), induced significant morphological changes and promoted agglutination of *E. coli*, *P. aeruginosa* and *Aeromonas hydrophila*. The activity of LJ-hep2_(66–86)_ against *E. coli*, *P. aeruginosa* and *A. hydrophila* was stable and remained active when heated for 30 min. In addition, LJ-hep2_(66–86)_ exhibited no cytotoxicity to the mammalian cell line HEK293T and fish cell lines (EPC and ZF4). In vivo study showed that LJ-hep2_(66–86)_ could improve the survival rate of marine medaka (*Oryzias melastigma*) by about 40% under the challenge of *A. hydrophila*, indicating its immunoprotective function. Taken together, both rLJ-hep2 and LJ-hep2_(66–86)_ have good prospects to be used as potential antimicrobial agents in aquaculture and medicine in the future.

## 1. Introduction

Aquaculture is an increasingly important industry, which provides high-quality aquatic food for human consumption. In recent years, aquaculture has become the main source of aquatic food [1]. Aquaculture production in the Asia-Pacific region accounts for more than 90% of global production, with China contributing approximately 60% [2]. However, the continuous expansion of aquaculture and serious marine pollution have led to the frequent occurrence of microbial diseases in aquaculture animals. The overuse of antibiotics to prevent or treat bacterial diseases not only easily leads to the emergence of drug-resistant bacteria, but also has a negative impact on the environmental ecosystem. In addition, the problem of drug residues in aquatic products has always been an important factor limiting the sustainable development of China’s fisheries. Therefore, in order to solve these problems caused by the use of antibiotics, there is an urgent need to develop alternative products of antibiotics that are efficient and environmentally friendly and make drug resistance more difficult. Antimicrobial peptides (AMPs) are a class of small-molecule peptides with broad-spectrum antimicrobial activities that exist in a variety of animals [3]. Compared with traditional antibiotics, AMPs do not easily induce pathogens to develop drug resistance and have immunomodulatory effects in vivo. Therefore, AMPs can be used as substitutes for antibiotics in the food animal industry, including aquaculture [4].

Fish live in complex aquatic environments and are constantly challenged by various pathogens. AMPs, as important components of innate immunity, are an important defensive weapon for fish against devastating diseases [5]. To date, the AMPs identified in fish mainly include defensins, cathelicidins, hepcidins, histone-family peptides, and piscidins [6]. Among these AMPs, hepcidins, also known as LEAP-1 (liver-expressed antimicrobial peptide), are small cysteine-rich peptides that were first identified in human blood ultrafiltrate and urine samples [7,8]. Hepcidin is initially produced as a pre-prohepcidin and ranges from 81–96 amino acids (aa), which will finally form a 19–31 aa mature peptide after enzymatic cleavage [6]. The function of hepcidins in multiple vertebrates has been investigated and reported to be involved in iron metabolism, inflammation, and clearance of invading pathogens [9]. Since the first fish hepcidin was reported in hybrid striped bass in 2002 [10], a large number of hepcidin isoforms have been found in numerous fish species. Unlike humans, where a single hepcidin gene exists, many teleost fish have more than two hepcidin genes, especially in Perciformes and Pleuronectiformes [11]. These hepcidin isoforms may exhibit distinct tissue-dependent expression patterns, indicating that they have different biological roles. Fish hepcidin isoforms are currently phylogenetically classified into two groups: HAMP1-type, with a single copy sharing a considerable degree of homology with mammalian counterparts and mainly present in liver tissue; HAMP2-type, which has multiple isoforms, wide tissue distribution and may exert antimicrobial effects [12,13,14]. A comprehensive analysis of the available studies revealed that the properties of hepcidin from each fish species are different (e.g., different antibacterial spectrum and the antiviral, antifungal, antiparasitic or antitumor activities), indicating that they have diverse functions and a wide range of potential application values [15]. Based on its multifaceted properties, fish hepcidin may be a potential candidate for the development of therapeutic agents in aquaculture and even medicine. Although a variety of hepcidins have been reported, there are relatively few studies on their functions and application values, compared with the sequence analysis and expression properties of hepcidin genes. An in-depth study on the discovered hepcidins to reveal their properties and functions will enrich the knowledge of hepcidin and develop hepcidin products with application values in the food animal industry and in medicine. In our previous study, a hepcidin-like gene (named Hepc2, GenBank No. AY604195) from Japanese seabass (*Lateolabrax japonicus*) was characterized [16]. Another study showed that, Hepc2 was highly expressed in the liver and kidney of *L. japonicus* and could be significantly up-regulated by LPS challenge [17]. Since Hepc2 of *L. japonicus* (designated as LJ-hep2) is a HAMP2-type hepcidin without the metal-binding conserved sequence (Q-S/I-H-L/I-S-L), it may play crucial roles in the immune defense system of fish, but the antimicrobial function and therapeutic application prospects have not been well studied.

In this study, the antimicrobial activity of LJ-hep2 was investigated using the synthetic peptide (LJ-hep2_(66–86)_) and recombinant protein obtained by *Pichia pastoris* eukaryotic expressing system (rLJ-hep2). To investigate the clinical application potential of LJ-hep2, we also examined the antimicrobial activity of LJ-hep2_(66–86)_ against several multidrug-resistant bacteria isolated from clinical samples. Furthermore, the time-killing kinetics assay, scanning electron microscope (SEM) analysis and bacterial agglutination assay were performed in vitro to analyze the antimicrobial features and mechanism of LJ-hep2_(66–86)_ against bacteria *Escherichia coli*, *Pseudomonas aeruginosa* and *Aeromonas hydrophila.* The thermal stability, cytotoxicity, sodium ion tolerance and the in vivo protective effect of LJ-hep2_(66–86)_ in marine medaka (*Oryzias melastigma*) under the challenge of aquatic pathogen *A. hydrophila* were further evaluated. The study will provide an experimental basis and technical support for the future application of LJ-hep2 in the aquaculture industry or in medicine development.

## 2. Results

### 2.1. The Chemically Synthesized LJ-hep2_(66–86)_ and Recombinant LJ-hep2 (rLJ-hep2) Obtained from P. pastoris Expression System

**Figure 1:**
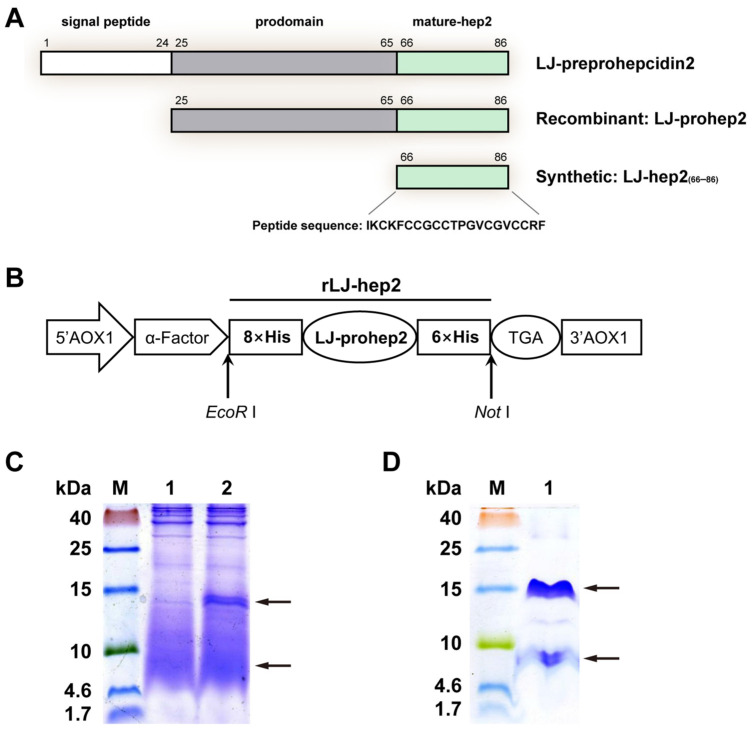
The preparation of synthetic LJ-hep2_(66–86)_ and eukaryotic expressed rLJ-hep2 peptides: (**A**) schematic diagram of primary structure of LJ-hep2. Different colors and amino acid number represent different structure domains; (**B**) the structure of recombinant plasmid pPIC9K-rLJ-hep2; (**C**) the eukaryotic expression of rLJ-hep2. Lane M: protein molecular standard; lane 1: negative control for rLJ-hep2 without methanol induction; lane 2: expression of rLJ-hep2 after methanol induction; and (**D**) purification of rLJ-hep2. Lane M: protein molecular standard; lane 1: purified rLJ-hep2.

The mature peptide of LJ-hep2 was chemically synthesized and named as LJ-hep2_(66–86)_ (Figure 1A). LJ-hep2_(66–86)_ consisted of 21 amino acid residues, with a theoretical molecular weight of 2.23 kDa, a theoretical pI of 8.53 and a total net charge of +3. The results from the mass spectrometry also showed that the observed molecular weight of LJ-hep2_(66–86)_ was 2230.05 Da (Figure S1, Supplementary Materials). The recombinant precursor protein of LJ-hep2 (rLJ-hep2) was successfully expressed in *P. pastoris* eukaryotic expression system after 24 h of methanol induction (Figure 1). After purification using affinity chromatography, two major bands with a molecular mass of about 8 kDa and 15 kDa were observed in Tricine-SDS-PAGE gel (Figure 1D), that corresponded with the predicted size of rLJ-hep2 monomer (8.8 kDa) and the dimer (17.6 kDa). The mass spectrometry analysis showed that both bands were the target protein rLJ-hep2 (Figure S2).

### 2.2. Antimicrobial Activity of LJ-hep2_(66–86)_ and rLJ-hep2

After serial dilution in sterile deionized water, the antimicrobial and bactericidal activities of LJ-hep2_(66–86)_ and rLJ-hep2 were examined using a variety of microbial strains. The minimum inhibitory concentration (MIC) and minimum bactericidal concentration (MBC) values obtained are summarized in Table 1. LJ-hep2_(66–86)_ displayed a broad antimicrobial spectrum, exerting potent activity against gram-negative bacteria (*Shigella flexneri*, *Pseudomonas fluorescens*, *Pseudomonas stutzeri*, *E. coli* and *P. aeruginosa*) and gram-positive bacteria (*Staphylococcus epidermidis*, *Corynebacterium glutamicum* and *Bacillus subtilis*), with MIC values ranging from 1.5–12 μM and MBC values lower than 12 μM. LJ-hep2_(66–86)_ showed relatively weak antimicrobial activity against gram-negative bacteria *A. hydrophila*, *Edwardsiella tarda* and *Aeromonas sobria*, with MIC values of 24–48 μM and MBC values of 24–48 μM. Compared with synthetic LJ-hep2_(66–86)_, rLJ-hep2 displayed a narrower antimicrobial spectrum to gram-negative bacteria, only exhibited antimicrobial activity against *S. flexneri* and *P. fluorescens*, but did not inhibit the growth of the other gram-negative bacteria tested in the present study. rLJ-hep2 showed strong antimicrobial activity against two tested gram-positive bacteria *S. epidermidis*, *C. glutamicum*, with MIC values of 1.5–3 μM and MBC values ranging from 1.5–12 μM, but showed relatively weak antimicrobial activity against *B. subtilis*, with MIC values of 24–48 μM. In addition, both LJ-hep2_(66–86)_ and rLJ-hep2 could inhibit the growth of a fungi *Cryptococcus neoformans*, with MIC values of 12–24 μM. We also evaluated the antimicrobial activity of LJ-hep2_(66–86)_ against several multidrug-resistant bacteria isolated from clinical samples, including *Acinetobacter baumannii*, *E. coli*, *P. aeruginosa*, *Klebsiella pneumoniae* and *Enterococcus faecium*. As shown in Table 2, LJ-hep2_(66–86)_ exerted potent activity against most of the tested multidrug-resistant bacteria, with MIC values ranging from 1.5–12 μM and MBC values lower than 24 μM.

### 2.3. Killing Kinetics of LJ-hep2_(66–86)_

**Figure 2:**
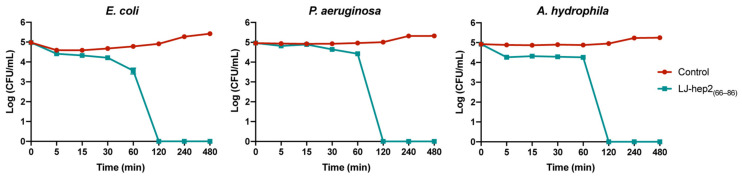
Time-killing kinetic curves of bacteria treated with LJ-hep2_(66–86)_ at a concentration of 2 × MBC (*E. coli*: 24 μM, *P. aeruginosa*: 24 μM and *A. hydrophila*: 96 μM). Bacteria treated without LJ-hep2_(66–86)_ were set as control groups.

The bacteria *E. coli*, *P. aeruginosa* and *A. hydrophila* were selected for time-killing kinetics assays to evaluate the bactericidal efficiency of LJ-hep2_(66–86)_. As shown in Figure 2, when incubated with 24 μM of LJ-hep2_(66–86)_, the viability of *E. coli* decreased continuously, and all microbes were killed after 2 h of incubation. The survival rate of *P. aeruginosa* decreased inconspicuously within 15 min, then decreased rapidly, and all microbes were killed after incubated with 24 μM of LJ-hep2_(66–86)_ for 2 h. 96 μM of LJ-hep2_(66–86)_ rapidly killed about 80% of *A. hydrophila* in 5 min and eliminated all microbes after 2 h.

### 2.4. LJ-hep2_(66–86)_ Induces Morphological Changes in Bacteria

**Figure 3:**
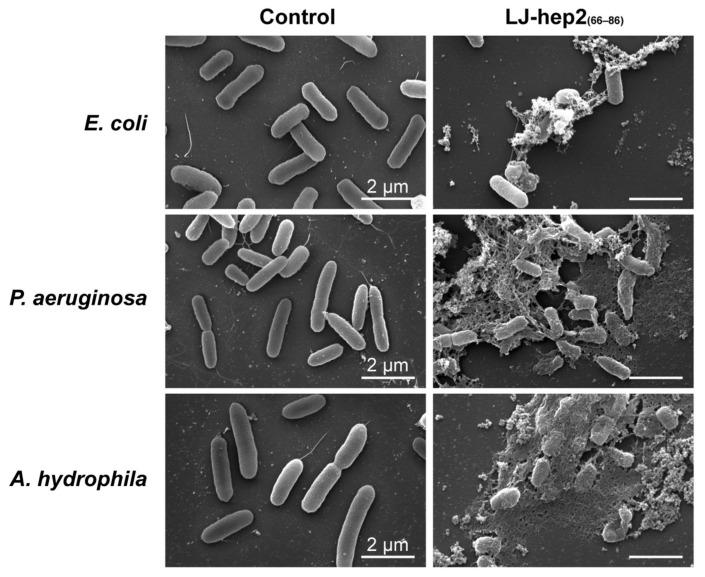
Morphology of bacteria treated by LJ-hep2_(66–86)_ observed by scanning electron microscopy (SEM). Microbial cells were suspended in culture media supplemented with PBS (Control) or LJ-hep2_(66–86)_ at a concentration of 2 × MBC (*E. coli*: 24 μM, *P. aeruginosa*: 24 μM and *A. hydrophila*: 96 μM), and observed by SEM.

In order to investigate the antibacterial mechanism of LJ-hep2_(66–86)_, the effect of LJ-hep2_(66–86)_ on bacterial surface structure was observed using SEM. LJ-hep2_(66–86)_ at concentrations of 2 × MBC (24 μM, 24 μM and 96 μM, respectively) were incubated with *E. coli*, *P. aeruginosa* and *A. hydrophila* for 1 h. Compared with untreated control groups, the LJ-hep2_(66–86)_-treated microbes showed cell shrinkage, destruction of membrane integrity, appearance of collapsed architecture and massive leakage of cellular contents (Figure 3).

### 2.5. LJ-hep2_(66–86)_ Promotes the Agglutination of Bacteria

**Figure 4:**
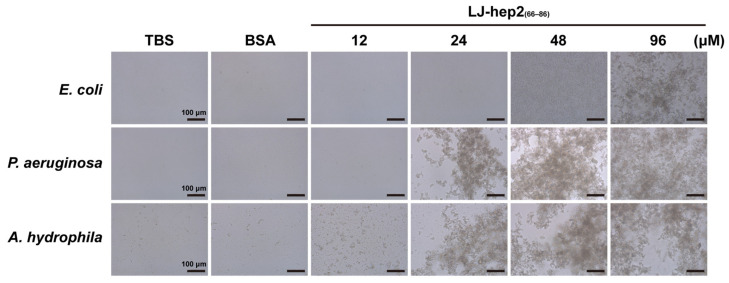
Agglutination activity of LJ-hep2_(66–86)_ against bacteria. Microbial cells were incubated with LJ-hep2_(66–86)_ peptide at final concentrations of 12, 24, 48 and 96 μM. TBS buffer and BSA (214 μg/mL) were used as control groups.

The results of bacterial agglutination assay showed that LJ-hep2_(66–86)_ had an agglutination-promoting effect on the three tested microbes, *E. coli*, *P. aeruginosa* and *A. hydrophila* (Figure 4). The microbes did not agglutinate at low concentrations of LJ-hep2_(66–86)_, and the agglutination phenomenon appeared obviously with the increase of peptide concentration. The minimum peptide concentration to induce agglutination differed among the three strains. *E. coli* required at least 96 μM of LJ-hep2_(66–86)_ to agglutinate, while *P. aeruginosa* and *A. hydrophila* microbes agglutinated at 24 μM of LJ-hep2_(66–86)_. The control groups (TBS buffer and BSA) failed to induce agglutination in the three tested microbes.

### 2.6. The Antimicrobial Activity of LJ-hep2_(66–86)_ Exhibits Thermal Stability

**Figure 5:**
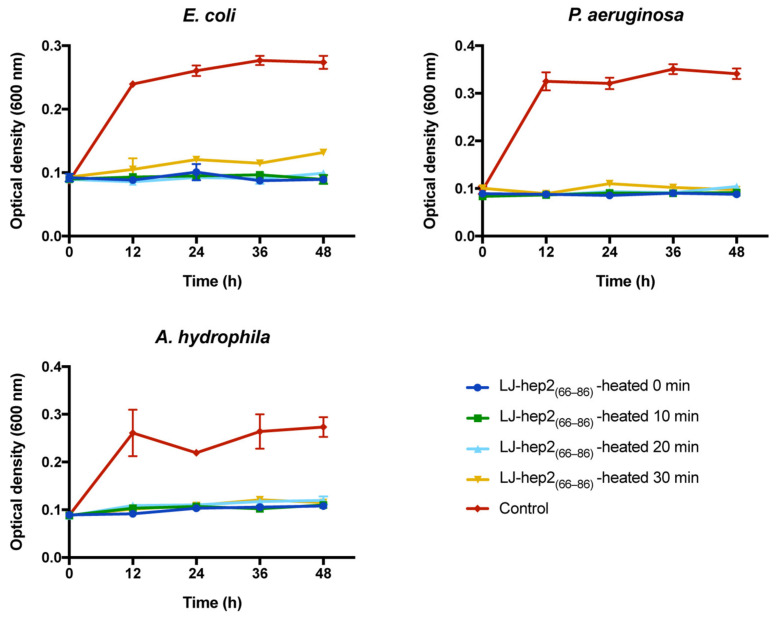
Thermal stability of LJ-hep2_(66–86)_. *E. coli*, *P. aeruginosa* and *A. hydrophila* were treated with heated LJ-hep2_(66–86)_ at a concentration of 2 × MBC. Bacteria treated without LJ-hep2_(66–86)_ were set as control groups.

Thermal stability analysis results (Figure 5) showed that the growth inhibitory effect of heated LJ-hep2_(66–86)_ on the three tested strains was no different from that of the unheated peptide, which indicated that the antimicrobial activity of LJ-hep2_(66–86)_ exhibited good thermal stability.

### 2.7. LJ-hep2_(66–86)_ Shows No Cytotoxicity

**Figure 6:**
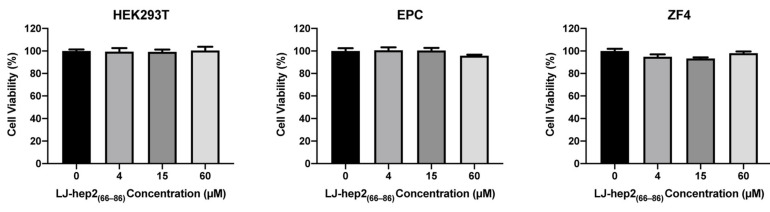
In vitro cytotoxicity of LJ-hep2_(66–86)_. The cytotoxic effects of LJ-hep2_(66–86)_ on HEK293T, EPC, and ZF4 were determined by the MTS method. Data are presented as mean ± standard errors of mean (*n* = 5). The statistical analysis was performed by one-way ANOVA followed by the Dunnett post hoc test.

The in vitro cytotoxic effect of LJ-hep2_(66–86)_ was evaluated using mammalian cell line HEK293T and fish cell lines (EPC and ZF4). As shown in Figure 6, the viability of cells incubated with serial concentrations of LJ-hep2_(66–86)_ (4, 15 and 60 μM) was not significantly different than the untreated cells. LJ-hep2_(66–86)_ exhibited no cytotoxic effect on mammalian and fish cells.

### 2.8. High Sodium Ion Concentration Impairs the Antimicrobial Activity of LJ-hep2_(66–86)_ against A. hydrophila

**Figure 7:**
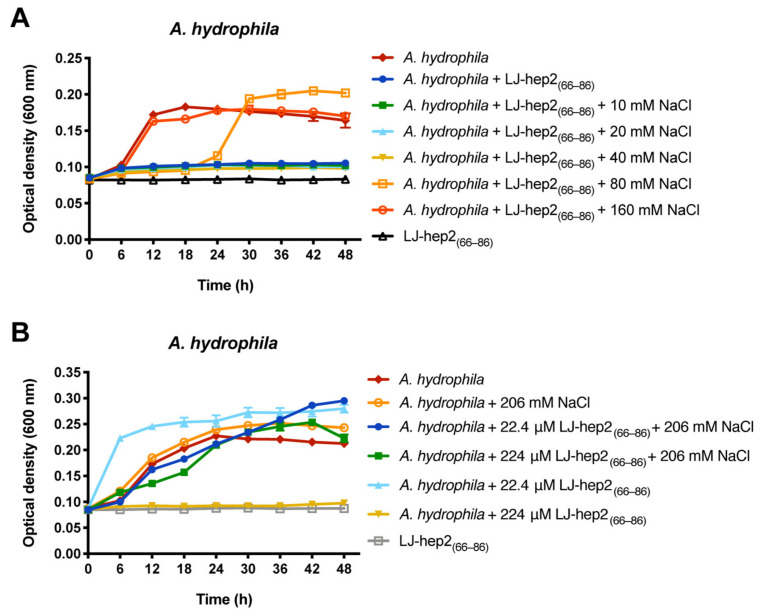
Sodium ion tolerance of LJ-hep2_(66–86)_: (**A**) The antimicrobial activity of LJ-hep2_(66–86)_ against *A. hydrophila* under various concentrations of NaCl. *A. hydrophila* suspension treated without LJ-hep2_(66–86)_ and LJ-hep2_(66–86)_ dilution without *A. hydrophila* were set as control groups; and (**B**) the antimicrobial activity of LJ-hep2_(66–86)_ against *A. hydrophila* under fish saline condition (206 mM NaCl).

The antimicrobial activity of LJ-hep2_(66–86)_ against *A. hydrophila* was evaluated in the presence of serial concentrations of NaCl (10, 20, 40, 80 and 160 mM), and the result showed that high concentrations of NaCl inhibited the antimicrobial activity of LJ-hep2_(66–86)_ against *A. hydrophila* (Figure 7A). LJ-hep2_(66–86)_ lost its bactericidal activity against *A. hydrophila*, when the concentration of NaCl was higher than 80 mM.

### 2.9. LJ-hep2_(66–86)_ Improves the Survival of O. melastigma in A. hydrophila Infection

**Figure 8:**
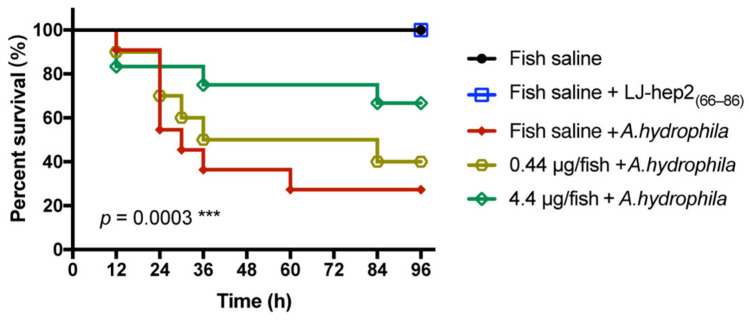
In vivo protective effect of LJ-hep2_(66–86)_ on *A. hydrophila*-infected *O*. *melastigma*. Fish were injected with *A. hydrophila* at 10^6^ CFU/fish and LJ-hep2_(66–86)_ at doses of 0.44 μg/fish and 4.4 μg/fish (*n* = 10–12 fish/group). The survival curve was analyzed using the Kaplan-Meier log-rank test. *** *p* < 0.001 indicated a significant difference.

In order to evaluate the in vivo protective effect of LJ-hep2_(66–86)_ on *A. hydrophila*-infected *O. melastigma*, we first investigated the feasibility of co-injection of LJ-hep2_(66–86)_ and *A. hydrophila* under fish saline (206 mM NaCl) conditions. The LJ-hep2_(66–86)_ peptide was injected into marine medaka at doses of 0.44 μg/fish and 4.4 μg/fish, and the final concentrations of the peptide were 22.4 μM and 224 μM, respectively. Therefore, the growth status of *A. hydrophila* was detected after incubation with 22.4 μM or 224 μM of LJ-hep2_(66–86)_ in fish saline. The results showed that, at either 22.4 μM or 224 μM, LJ-hep2_(66–86)_ could not inhibit the growth of *A. hydrophila* under fish saline conditions (Figure 7B). In addition, the growth status of *A. hydrophila* in fish saline conditions was no different than the control *A. hydrophila* suspension group. Based on these in vitro results, marine medaka were co-injected with *A. hydrophila* at 10^6^ CFU/fish and LJ-hep2_(66–86)_ at 0.44 μg/fish or 4.4 μg/fish. As shown in Figure 8, the survival rate of the *A. hydrophila*-infected control group decreased to around 27% at 60 h post-injection, while both the operating control group and the protein control group (without *A. hydrophila* challenged) showed 100% survival rates. The survival rate of the treatment group injected with LJ-hep2_(66–86)_ at 0.44 μg/fish was 40% at 96 h post-injection, while the survival rate of the other treatment group injected with LJ-hep2_(66–86)_ at 4.4 μg/fish was around 67%, which was increased by 40% compared to the *A. hydrophila*-infected control group. These results indicated that LJ-hep2_(66–86)_ could significantly improve the survival rate of *A. hydrophila*-infected marine medaka.

## 3. Discussion

Hepcidin, encode by HAMP gene in mammals, has two major functions: regulating iron homeostasis and innate immunity. Since hepcidin was identified as a hormonal regulator of iron metabolism, much attention has been paid to characterize the role and mechanism of hepcidin in iron-dysregulation diseases with the aim of translating into hepcidin-targeted therapy [18]. Several studies in animal models have shown that restoring appropriate hepcidin levels can be used to treat iron-dysregulation diseases, such as hereditary hemochromatosis and iron-refractory iron deficiency anemia [19,20]. In contrast, many hepcidin genes identified in fish species have demonstrated their major functions in antimicrobial activities and immune regulatory effects [15]. Unlike humans with only one hepcidin gene, or mice with two isoforms, most fish species have multiple copies of hepcidin, especially the HAMP2-type hepcidin, such as the 7 hepcidin isoforms cloned from black porgy (*Acanthopagrus schlegelii*) [21]. The distinct tissue-dependent expression pattern of these hepcidin isoforms may be related to various factors, such as the diversity of fish habitats and aquatic environments, and the degree of pathogens invasion [11,22]. Most studies have concluded that polymorphism in fish hepcidin is required to prevent the invasion of pathogenic microorganisms. Fish hepcidins have enormous potential for further development as antimicrobial agents. For example, a study of a recombinant hepcidin product from grass carp (*Ctenopharyngodon idellus*) demonstrated its immunomodulatory effects and indicated a potential in controlling bacterial infections in aquaculture [23]. Therefore, the investigation on the biological activities of fish hepcidin products could provide more possibilities for the application of effective antimicrobial agents in various industrial fields in the future.

The proposal of applying hepcidin as antimicrobial agents in industry, such as animal husbandry and aquaculture requires the development of economically friendly methods to produce these oligopeptides. As cysteine-rich small peptides, hepcidins are biologically active when they are correctly folded to form intramolecular disulfide bonds and stabilize a general secondary hairpin structure with β-sheet [24]. However, it is still difficult to artificially produce the correctly folded, cysteine-rich, cationic hepcidin. At present, AMP products are mainly obtained through chemical synthesis and genetic engineering expression. Genetic engineering expression is an effective way to obtain a large number of highly expressed products, which is beneficial to the large-scale development and utilization of AMPs. However, several studies have reported that with hepcidin, it is not easy to directly obtain the expression product with high activity. Some successfully expressed hepcidins were expressed in the form of fusion proteins [23,25], or expressed in inclusion bodies of *E. coli* [26,27,28], which requires a complex refolding process, and is not conducive to large-scale production. In addition, there are some soluble-expressed hepcidins that are obtained easily but have poor antimicrobial activities, such as the recombinant human hepcidin expressed in *P. pastoris* [29] and the soluble recombinant EC-hepcidin3 expressed in *E. coli* [30]. According to previous studies, hepcidin generally functions as a mature peptide after hydrolysis of the prodomain in organisms [31], but the prodomain plays an essential role in the correct folding of many proteins to which they are attached [32]. In addition, it is generally believed that the His-tags have negligible effects on the activity of recombinant proteins, and it is conducive to obtain the desired protein purity and amount with two His-tags when purifying the target protein. Therefore, in this study, a soluble recombinant fish hepcidin precursor protein (with prodomain and mature peptide) containing two His-tags at the N-terminus and C-terminus, respectively, was successfully expressed in *P. pastoris* and exhibited potent activities against several gram-positive and gram-negative bacteria and fungi. Considering the antimicrobial spectrum and the capability to be easily obtained by large-scale fermentation, the recombinant expression product rLJ-hep2 could be widely used for future applications in animal husbandry or aquaculture, such as being used as a dietary supplement. In addition, previous studies in our laboratory found that several chemically synthesized mature peptides of fish hepcidin (e.g., PC-hepc, AS-hepc2 and AS-hepc6) exerted broad-spectrum antimicrobial activity, including for important clinical pathogens and aquatic pathogens, such as *S. aureus*, *E. coli* and *A. hydrophila* [13,33]. Similarly, the synthetic LJ-hep2_(66–86)_ in our current study exerted potent antimicrobial activities and was stable and remained functional under heat-stress conditions. In particular, the potent antimicrobial activities of LJ-hep2_(66–86)_ against clinical and aquatic bacterial pathogens *E. coli*, *P. aeruginosa* and *A. hydrophila*, as well as several multi-drug resistant bacteria indicated its high application value in aquaculture and clinical medicine development. The results of antibacterial spectrum indicated that synthetic mature peptide LJ-hep2_(66–86)_ exhibited better antimicrobial activities than recombinant protein rLJ-hep2. We speculate that the presence of prodomain in rLJ-hep2 might limit the antimicrobial activity of the mature peptide due to its spatial conformation, ultimately resulting in a lower activity of the recombinant peptide than the synthetic mature peptide. The effects of prodomain on the function of hepcidins would be worth investigating in future work.

The growing emergence and spread of multidrug-resistant bacteria have become a major concern after decades of antibiotics use in the prevention and treatment of pathogenic bacteria infections. Several common clinical bacterial pathogens, such as *A. baumannii*, *E. coli*, *P. aeruginosa* and *K. pneumoniae* have acquired resistance to a wide range of antibiotics, posing a significant threat to public health [34]. Our current study revealed that LJ-hep2_(66–86)_ possessed strong antimicrobial activities against these multi-drug resistant bacteria isolated from clinical samples. This result is consistent with the previous findings that the peptides Sp-NPFin [35] from *Scylla paramamosain* and AS-hepc3_(48–56)_ [36] from *A. schlegelii* effectively inhibited the growth of several multi-drug resistant bacteria. Currently, AMPs are being explored as alternative therapy to tackle infections caused by multidrug-resistant bacteria [37]. Therefore, the antimicrobial activity of LJ-hep2_(66–86)_ against multidrug-resistant bacteria will provide more possibilities with regard to the exploration of potential anti-drug resistant bacterial drugs.

As essential effectors in innate immunity, AMPs play critical roles in the host to combat invading pathogens [3]. Our study showed LJ-hep2_(66–86)_ protected *O. melastigma* from *A. hydrophila* challenges, and increased the survival rate by 40%. Similar immune protection was also evaluated in many other fish hepcidins. Almost without exception, SmHep1P and SmHep2P from *Scophthalmus maximus* [38], LrHep from *Labeo rohita* [39], Tf-Hep from *Trachidermus fasciatus* [40] and AN-hepc from *Amatitlania nigrofasciata* [41] significantly improved the survival rate of bacteria-infected fish. Accumulating evidence suggests that AMPs play an immunomodulatory role, mainly by suppressing pathogen-induced detrimental pro-inflammatory responses, ultimately reducing fish mortality [42]. Even in the absence of pathogens, fish hepcidin can also function as an immunomodulator to activate immune responses [43]. Under normal physiological conditions, the osmotic pressure of the internal environment of marine teleost is maintained at approximately 350–400 mOsmol/L, which is equivalent to a salinity of approximately 10–15 [44,45]. Na^+^, one of the major dissolved ions, is maintained at approximately 160–180 mM [46], which is well above the sodium tolerance range of LJ-hep2_(66–86)_ in our present study. Therefore, the in vivo protective effect of LJ-hep2_(66–86)_ might not be through its antimicrobial activity but might be related to immunomodulatory functions. The regulatory role of hepcidins on the host immune response against infected bacteria has been well studied in fish. Overexpression of tilapia hepcidins (TH1-5 and TH2-3) in transgenic zebrafish infected with *Vibrio* significantly reduced the bacterial load in tissues, regulated immune-related genes and effectively enhanced the resistance to pathogen infection [47,48]. In addition to hepcidins, other types of AMPs, such as SpHyasrtatin [49] and Sparanegtin [50] from mud crab *S. paramamosain* may also protect crabs against pathogenic infections by exerting immunomodulatory effects rather than direct antimicrobial activities. Taken together, LJ-hep2_(66–86)_ may act as an important immune-protective factor in response to invading pathogens.

In summary, a recombinant product of rLJ-hep2 from *L. japonicus* was successfully obtained through *P. pastoris* expression system. The synthetic mature peptide LJ-hep2_(66–86)_ showed potent activity against a variety of bacteria and fungi. In addition, LJ-hep2_(66–86)_ exerted potent antimicrobial activity against multidrug-resistant bacteria. The antimicrobial mechanism of LJ-hep2_(66–86)_ might be related to its destruction of bacterial membrane structure and promotion of bacterial agglutination. Moreover, LJ-hep2_(66–86)_ showed no cytotoxic effect on mammalian and fish cell lines and could improve the survival rate of *O. melastigma* under *A. hydrophila* challenge. Taken together, this study revealed the antimicrobial activities of rLJ-hep2 and LJ-hep2_(66–86)_. The potent broad-spectrum antimicrobial activity and in vivo protective effect provides an important basis for the development and application of these two hepcidin products as antimicrobial agents.

## 4. Materials and Methods

### 4.1. Animals, Cell Lines and Microorganisms

Marine medaka (*O. melastigma*) were bred and reared in the State Key Laboratory of Marine Environmental Science (Xiamen University). The fish selected for this study were 3 months old and had reached sexual maturity. All animal procedures were carried out in strict accordance with the guidelines of Xiamen University.

HEK293T (ATCC^®^CRL-3216) cell line was purchased from the National Collection of Authenticated Cell Cultures (Shanghai, China) and cultured in DMEM supplemented with 10% fetal bovine serum (FBS, Gibco) at 37 °C and 5% CO_2_ in air atmosphere. ZF4, a zebrafish (*Danio rerio*) embryonic fibroblast cell line, was purchased from the China Zebrafish Resource Center and cultured in DMEM-F12 (1:1) supplemented with 10% FBS at 28 °C and 5% CO_2_ in air atmosphere. EPC (Epithelioma Papulosum Cyprini, ATCC^®^CRL 2872) cell line was cultured in Leibovitz’s L-15 Medium (HyClone) supplemented with 10% FBS and 5 mM 4-(2-hydroxyethyl)-1-piperazineethanesulfonic acid (HEPES, Sigma-Aldrich) at 26 °C. The commercially available strains were purchased from China General Microbiological Culture Collection Center (CGMCC), including *S. flexneri* (CGMCC 1.1868), *P. fluorescens* (CGMCC 1.3202), *P. stutzeri* (CGMCC 1.1803), *E. coli* (CGMCC 1.2389), *P. aeruginosa* (CGMCC 1.2421), *A. hydrophila* (CGMCC 1.2017), *S. epidermidis* (CGMCC 1.4260), *C. glutamicum* (CGMCC 1.1886), *B. subtilis* (CGMCC 1.3358) and *C. neoformans* (CGMCC 2.1563). *P. pastoris* GS115 was purchased from Invitrogen (USA). *E. tarda* and *A. sobria* isolated from diseased fish were kindly provided by Freshwater Fisheries Research Institute of Fujian. Multidrug-resistant bacteria strains isolated from clinical samples, including *A. baumannii* (QZ18050, QZ18055), *E. coli* (QZ18109, QZ18110), *P. aeruginosa* (QZ19124, QZ19125), *K. pneumoniae* (QZ18106, QZ18107) and *E. faecium* (QZ18080, QZ18081) were kindly provided by the Second Affiliated Hospital of Fujian Medical University (Quanzhou, Fujian, China). The bacterial strains were cultured in nutrient broth (OXOID, UK) agar according to their optimum growth temperature, and the fungal strains, including *C. neoformans* and *P. pastoris* were cultured in yeast extract peptone dextrose (OXOID, UK) agar at 28 °C.

### 4.2. Expression and Purification of Recombinant LJ-hep2 (rLJ-hep2) in the Yeast P. pastoris

The recombinant product of LJ-hepc2 in this study consisted of an N-terminal 8 × His-tag followed by LJ-hep2 precursor protein (LJ-prohepc2, with prodomain and mature peptide), as well as a C-terminal 6 × His-tag (Figure 1A,B). The sequence encoding LJ-prohepc2 was sent to GenScript Biotech (Nanjing, China) to optimize the codons and inserted into the *Pichia* expression vector pPIC9K (Invitrogen, Waltham, MA, USA), with two His-tags linked for subsequent affinity purification. The resulting recombinant plasmid pPIC9K-rLJ-hep2 was linearized using Sac I. The linearized plasmid was transformed to *P. pastoris* GS115 cells using electroporation. The positive clone of GS115/pPIC9K-rLJ-hep2 was chosen for large-scale recombinant expression induced with 0.5% methanol for 24 h, as described previously [51]. The supernatant containing the secreted rLJ-hep2 peptide was collected by centrifugation at 12,000× *g* for 30 min at 4 °C, dialyzed in sodium phosphate buffer (50 mM NaPB, 50 mM NaCl, pH 8.0), then loaded to a HisTrap^TM^ FF crude column (GE Healthcare Life Sciences, Chicago, IL, USA) and purified using ÄKTA Pure system (GE Healthcare Life Sciences, Chicago, IL, USA). The purified rLJ-hep2 was finally dialyzed in deionized water and analyzed by the Tricine-SDS-PAGE. The specific bands were confirmed by a mass spectrometer (timsTOF Pro, Bruker Daltonik GmbH, Bremen, Germany). The purified rLJ-hep2 peptide was sterilized by filtration using a 0.2 μm low protein binding syringe filter (PALL Corporation, New York, NY, USA), followed by measurement of protein concentration using a Bradford assay kit (Beyotime Biotechnology, Shanghai, China), and finally stored at −80 °C before use.

### 4.3. Synthesis of the Mature Peptide LJ-hep2_(66–86)_

According to the organization analysis of LJ-hep2 gene in previous study [16], the predicted mature peptide is from amino acids 66 to 86 and consist of 21 amino acid residues (theoretical molecular weight 2.23 kDa) shown as follows: IKCKFCCGCCTPGVCGVCCRF (Figure 1A). The mature-hep2 (named as LJ-hep2_(66–86)_) used in this study was chemically synthesized, purified via HPLC and verified via mass spectrometry by Sangon Biotech (Shanghai, China). The purity of synthetic LJ-hep2_(66–86)_ was over 95% and could be dissolved in sterile deionized water. The powdered peptide was stored at −80 °C until use.

### 4.4. Antimicrobial Assays

The microorganisms used to test the antimicrobial activities of LJ-hep2_(66–86)_ and rLJ-hep2 were harvested during their logarithmic growth phase, washed with 10 mM NaPB (pH 7.4), adjusted to 3.3 × 10^4^ CFU/mL (bacteria and yeasts) in 10 mM NaPB supplemented with 40% Mueller–Hilton broth, and then added an equal volume of serially diluted peptides. The MIC and MBC of peptide were determined in triplicate on separated occasions using liquid growth inhibition assays as described previously [49].

### 4.5. Time-Killing Kinetics

The time-killing kinetics assay was performed as previously described [35,49]. The bacteria *E. coli*, *P. aeruginosa* and *A. hydrophila* were harvested and adjusted to 3.3 × 10^4^ CFU/mL as described for the antimicrobial assay. LJ-hep2_(66–86)_ was incubated with bacteria at a concentration of 2 × MBC. The treated bacterial suspensions were sampled, diluted, and plated on nutrition broth agar plates at different time points (5 min, 15 min, 30 min, 60 min, 120 min, 240 min and 480 min). After incubation overnight (*E. coli* and *P. aeruginosa* at 37 °C, *A. hydrophila* at 28 °C), the surviving colonies were counted. The experiments were conducted in triplicate.

### 4.6. Scanning Electron Microscope (SEM) Analysis

The bacteria *E. coli*, *P. aeruginosa* and *A. hydrophila* were prepared as described in the antimicrobial assay and adjusted to 5 × 10^7^ CFU/mL. The peptide LJ-hep2_(66–86)_ was added to bacterial suspensions at a concentration of 2 × MBC. After incubation for 1 h, the bacteria cells were collected by centrifugation at 3000× *g* for 5 min, then fixed with pre-cooled 2.5% glutaraldehyde at 4 °C for 2 h. The cells were deposited on a glass slide covered with polylysine after washing with PBS (phosphate buffered saline, pH 7.4), then dehydrated using a graded series of ethanol (30%, 50%, 70%, 80%, 95%, and 100%) and lyophilized in a critical point dryer (EM CPD300, Leica, Wetzlar, Germany). Finally, the cells were gold-coated and observed with a scanning electron microscope (FEI Quanta 650 FEG, Thermo Fisher, Hillsboro, OR, USA).

### 4.7. Bacterial Agglutination Assay

The bacteria *E. coli*, *P. aeruginosa* and *A. hydrophila* were harvested during their logarithmic growth phase, washed 3 times with TBS buffer (pH 7.4) and adjusted to 10^8^ CFU/mL. Afterwards, the bacteria were added to the equal volume of the serial diluted LJ-hep2_(66–86)_ peptide (with final concentrations of 12, 24, 48 and 96 μM) in 96-well polystyrene flat-bottom plates (NEST, Wuxi, China). TBS buffer and BSA (214 μg/mL) were used as control groups. After incubation at room temperature for 1 h, the agglutination results were observed under a microscope (Zeiss, Oberkochen, Germany). Each treatment had three biological replicates. The independent experiment was performed in triplicate.

### 4.8. Thermal Stability Analysis

In order to analyze the thermal stability of antimicrobial activity of LJ-hep2_(66–86)_, the bacteria *E. coli*, *P. aeruginosa* and *A. hydrophila* were incubated with heated LJ-hep2_(66–86)_ at a concentration of 2 × MBC. Briefly, the three bacteria were prepared as described in the antimicrobial assay. The diluted LJ-hep2_(66–86)_ peptides were heated by boiling water bath for 0, 10, 20 and 30 min. The bacterial suspensions were added to the equal volume of the heated LJ-hep2_(66–86)_ peptide (with final concentrations of 2 × MBC) in 96-well polystyrene flat-bottom plates (NEST, Wuxi, China) and incubated at optimum growth temperature (*E. coli* and *P. aeruginosa* at 37 °C, *A. hydrophila* at 28 °C). The growth status of bacteria was monitored at 600 nm using a microplate reader (Infinite F200 PRO, TECAN, Männedorf, Switzerland) after incubation for 0, 12, 24, 36 and 48 h. Bacteria treated without LJ-hep2_(66–86)_ were set as control groups. Each treatment had three biological replicates, and three independent experiments were performed.

### 4.9. Cytotoxicity Assay

The cytotoxicity of LJ-hep2_(66–86)_ to mammalian or fish cells was evaluated using a microplate MTS assay. HEK293T, EPC and ZF4 cells were seeded at 10^4^ cells/well on a 96-well flat-bottomed plate (Thermo Fisher, Waltham, MA, USA) and cultured in their own conditions overnight before incubated with peptide. Then, the culture medium was replaced with fresh medium containing LJ-hep2_(66–86)_ peptide with the final concentrations of 0, 4, 15 and 60 μM. Cell viability was assessed using a CellTiter 96^®^ AQ_ueous_ Non-Radioactive Cell Proliferation Assay kit (Promega, Madison, WI, USA) after incubation for 24 h, as previously described [36]. Each treatment had five biological replicates, and three independent experiments were performed.

### 4.10. Sodium Ion Tolerance Analysis

The antimicrobial activity of LJ-hep2_(66–86)_ was analyzed under various concentrations of sodium ion. The tested microbe *A. hydrophila* was prepared as described in the antimicrobial assay, then incubated with LJ-hep2_(66–86)_ peptide (with final concentration of 2 × MBC) in a 96-well polystyrene flat-bottom plate (NEST, Wuxi, China). The treated bacterial suspensions were supplemented with various concentrations of NaCl (0, 10, 20, 40, 80 and 160 mM). The growth status of *A. hydrophila* was monitored at 600 nm using a microplate reader (Infinite F200 PRO, TECAN, Männedorf, Switzerland) after incubation at 28 °C for 0, 6, 12, 18, 24, 30, 36, 42 and 48 h. Bacterial suspension treated without LJ-hep2_(66–86)_ and LJ-hep2_(66–86)_ dilution without *A. hydrophila* were set as control groups. Each treatment had three biological replicates, and three independent experiments were performed. 

In order to facilitate the follow-up study on the in vivo protective effect of LJ-hep2_(66–86)_ on fish infected with *A. hydrophila*, the antimicrobial activity of LJ-hep2_(66–86)_ against *A. hydrophila* in fish saline (206 mM NaCl) was investigated as mentioned above. Seven groups were set up (*A. hydrophila* with 206 mM NaCl, 22.4 μM LJ-hep2_(66–86)_ treated *A. hydrophila* with or without 206 mM NaCl, 224 μM LJ-hep2_(66–86)_ treated *A. hydrophila* with or without 206 mM NaCl, *A. hydrophila* suspension and LJ-hep2_(66–86)_ dilution as two control groups). The growth status of *A. hydrophila* was also monitored after incubation for 0, 6, 12, 18, 24, 30, 36, 42 and 48 h.

### 4.11. Evaluation of the In Vivo Protective Effect of LJ-hep2_(66–86)_ on A. hydrophila-Infected O. melastigma

In the study, male marine medaka *O. melastigma* and the pathogenic bacteria *A. hydrophila* were selected to investigate the in vivo protective effect of LJ-hep2_(66–86)_. During the experiment period, the marine medaka were reared in glass aquariums with 5 L of artificial sea water at salinity 30 and a steady photoperiod of 14 h light /10 h dark. The fish were separated randomly into five groups (*n* =10–12 fish/group), and temporarily reared for 4 days before experiment. LJ-hep2_(66–86)_ and *A. hydrophila* were prepared in fish saline (206 mM NaCl) for the experiments. Briefly, *A. hydrophila* were harvested during their logarithmic growth phase, washed 3 times with fish saline and adjusted to 2.3 × 10^8^ CFU/mL. LJ-hep2_(66–86)_ was dissolved with sterile fish saline and adjusted to 100 μg/mL and 1000 μg/mL. The bacterial suspensions were mixed with equal volume of the diluted LJ-hep2_(66–86)_ (100 and 1000 μg/mL) and injected intraperitoneally into medaka immediately with 8.8 μL per fish. Thus, the two treated groups were injected with *A. hydrophila* at 10^6^ CFU/fish and LJ-hep2_(66–86)_ at 0.44 μg/fish and 4.4 μg/fish. At the same time, fish injected with *A. hydrophila* (10^6^ CFU/fish) were set up as *A. hydrophila*-infection controls; fish injected with LJ-hep2_(66–86)_ (4.4 μg/fish) were set up as protein controls; fish injected with fish saline were set up as operating controls. Fish mortality in each group was monitored and recorded at different time points (0, 12, 24, 36, 48, 60, 72, 84 and 96 h). The survival rate was calculated.

### 4.12. Statistical Analysis

Data were presented as means ± standard errors of mean. The statistical analysis of cytotoxicity assays was performed by one-way ANOVA followed by the Dunnett post hoc test to compare the cell viability of different concentrations of LJ-hep2_(66–86)_ treatment and control groups in IBM SPSS Statistics (version 22; IBM Corp., Armonk, NY, USA). The result of fish mortality was analyzed by the Kaplan–Meier Log-rank test in GraphPad Prism software (version 8; GraphPad Software Inc., San Diego, CA, USA). The graphs were drawn by GraphPad Prism 8 software and * *p* < 0.05 indicated a significant difference.

## Figures and Tables

**Table 1:** Antibacterial spectrum of rLJ-hep2 (recombinant protein) and LJ-hep2_(66–86)_ (chemically synthesized peptide).

Microorganisms	CGMCC No. ^a^	rLJ-hep2		LJ-hep2_(66–86)_	
		MIC (μM) ^b^	MBC (μM) ^b^	MIC (μM) ^b^	MBC (μM) ^b^
Gram-negative bacteria					
*Shigella flexneri*	1.1868	1.5–3	1.5–3	3–6	3–6
*Pseudomonas fluorescens*	1.3202	12–24	>48	6–12	6–12
*Pseudomonas stutzeri*	1.1803	>48	>48	1.5–3	3–6
*Escherichia coli*	1.2389	>48	>48	6–12	6–12
*Pseudomonas aeruginosa*	1.2421	>48	>48	6–12	6–12
*Aeromonas hydrophila*	1.2017	>48	>48	24–48	24–48
*Edwardsiella tarda*	_ ^c^	>48	>48	24–48	24–48
*Aeromonas sobria*	_ ^c^	>48	>48	24–48	24–48
Gram-positive bacteria					
*Staphylococcus epidermidis*	1.4260	1.5–3	1.5–3	6–12	6–12
*Corynebacterium glutamicum*	1.1886	1.5–3	6–12	3–6	3–6
*Bacillus subtilis*	1.3358	24–48	>48	6–12	6–12
Fungi					
*Cryptococcus neoformans*	2.1563	12–24	12–24	12–24	24–48

^a^ CGMCC No., China General Microbiological Culture Collection Number. ^b^ The MIC and MBC values are presented as the interval (A), (B): (A) is the highest concentration tested with visible microbial growth, while (B) is the lowest concentration without visible microbial growth (*n* = 3). ^c^ Bacteria isolated from diseased fish, provided by Freshwater Fisheries Research Institute of Fujian.

**Table 2:** Anti-drug resistant bacterial activities of chemically synthesized LJ-hep2_(66–86)_.

Multidrug-Resistant Bacteria	MIC (μM) ^a^	MBC (μM) ^a^
Gram-negative bacteria		
*Acinetobacter baumannii* (QZ18050)	1.5–3	3–6
*Acinetobacter baumannii* (QZ18055)	1.5–3	3–6
*Escherichia coli* (QZ18109)	3–6	3–6
*Escherichia coli* (QZ18110)	3–6	6–12
*Pseudomonas aeruginosa* (QZ19124)	6–12	12–24
*Pseudomonas aeruginosa* (QZ19125)	6–12	12–24
*Klebsiella pneumoniae* (QZ18106)	6–12	6–12
*Klebsiella pneumoniae* (QZ18107)	12–24	>48
Gram-positive bacteria		
*Enterococcus faecium* (QZ18080)	6–12	6–12
*Enterococcus faecium* (QZ18081)	6–12	6–12

^a^ The MIC and MBC values are presented as the interval (A), (B): (A) is the highest concentration tested with visible microbial growth, while (B) is the lowest concentration without visible microbial growth (*n* = 3).

## Data Availability

Not applicable.

## Supplementary Materials

The following supporting information can be downloaded at: https://www.mdpi.com/article/10.3390/md20100651/s1, Figure S1: The mass spectrometry analysis of the chemically synthesized peptide LJ-hep2_(66-86)_; Figure S2: The mass spectrometry analysis of the two bands of rLJ-hep2.

## Abbreviations

aa: amino acids; AMPs, antimicrobial peptides; CGMCC, China General Microbiological Culture Collection Center; FBS, fetal bovine serum; MBC, minimum bactericidal concentration; MIC, minimum inhibitory concentration; SEM, scanning electron microscope.
