# Supplementary material for: The Antimicrobial Peptide LJ-hep2 from Lateolabrax japonicus Exerting Activities against Multiple Pathogenic Bacteria and Immune Protection In Vivo
Source: Mar Drugs. 2022 Oct 20;20(10):651. doi: 10.3390/md20100651 (PMC9605627; doi:10.3390/md20100651)
Supplement: Supplementary file 1 [file marinedrugs-20-00651-s001.zip › marinedrugs-1956195-supplementary.pdf]

Supplementary Materials

The Antimicrobial Peptide LJ-hep2 from *Lateolabrax japonicus* Exerting Activities against Multiple Pathogenic Bacteria and Immune Protection In Vivo

Ruihao Gong [1,†], Zhe An [1,†], Weibin Zhang [1], Fangyi Chen [1,2,3] and Ke-Jian Wang [1,2,3,*]

[1] State Key Laboratory of Marine Environmental Science, College of Ocean & Earth Sciences, Xiamen University, Xiamen 361102, Fujian, China

[2] State-Province Joint Engineering Laboratory of Marine Bioproducts and Technology, College of Ocean & Earth Sciences, Xiamen University, Xiamen 361102, Fujian, China

[3] Fujian Innovation Research Institute for Marine Biological Antimicrobial Peptide Industrial Technology, College of Ocean & Earth Sciences, Xiamen University, Xiamen 361102, China

† These authors contributed equally to this work.

* Corresponding author: Ke-Jian Wang (K.-J. Wang)

College of Ocean & Earth Sciences, Xiamen University, Xiamen 361102, Fujian, PR China.

E-mail: wkjian@xmu.edu.cn

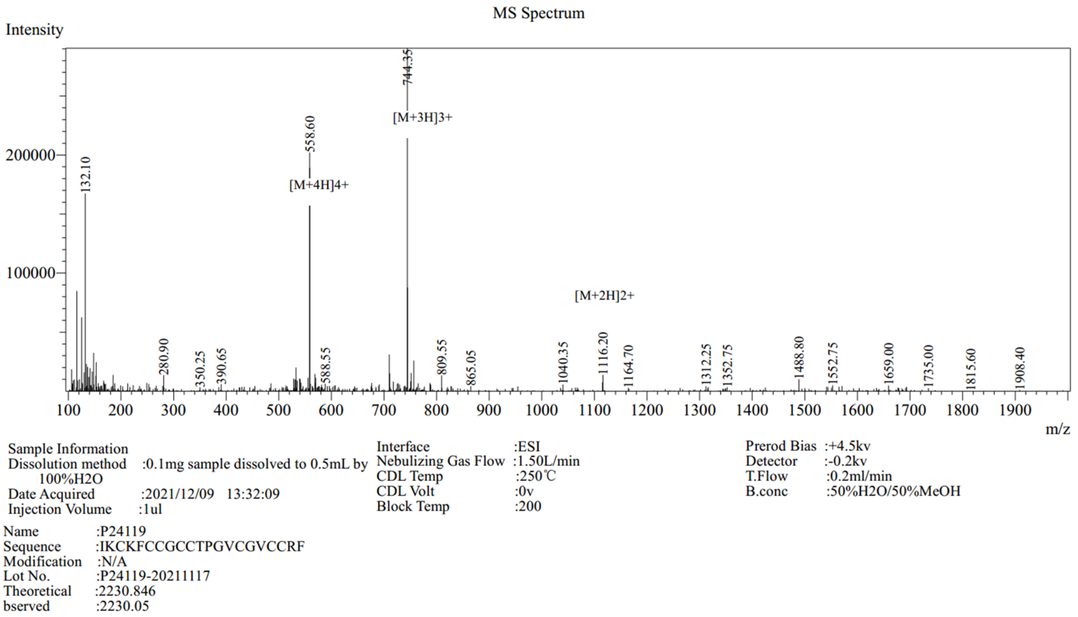

Figure S1. The mass spectrometry analysis of the chemically synthesized peptide LJ-hep2$_{(66–86)}$. The MS analysis result showed the information of the peptide, including the sequence, the theoretical and observed molecular weight.

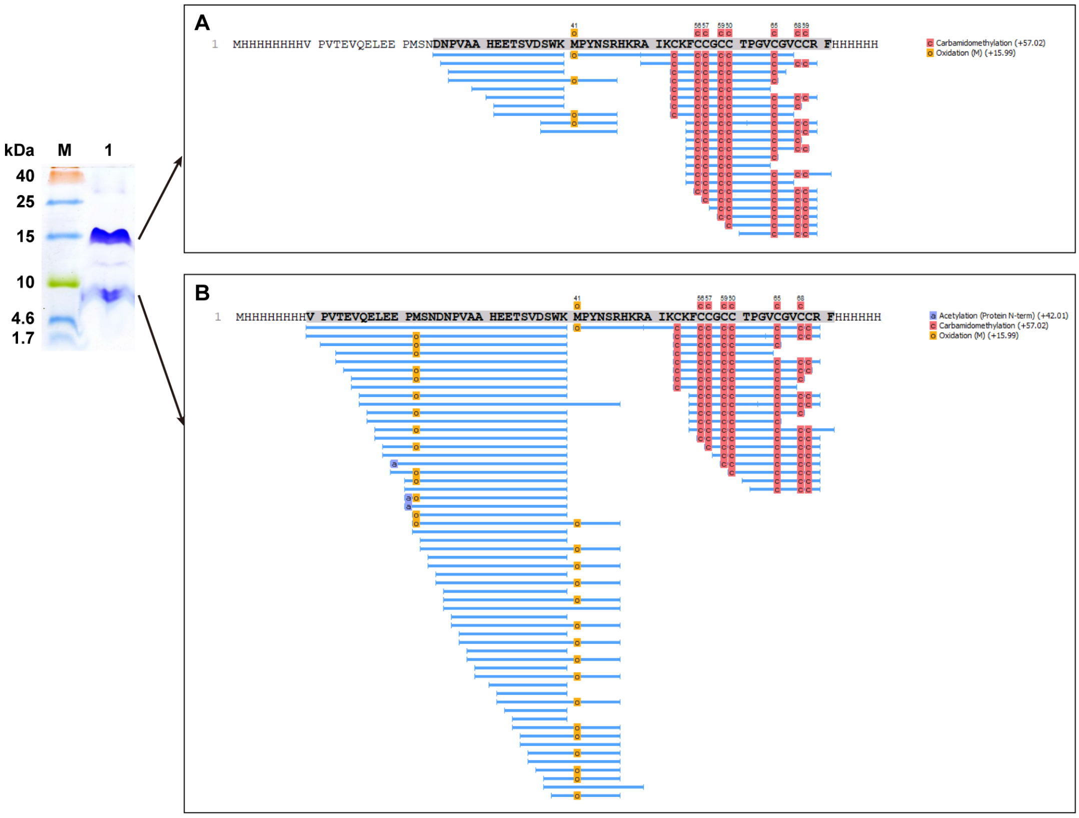

Figure S2. The mass spectrometry analysis of the two bands of rLJ-hep2. A: the coverage result of the peptide with molecular mass of about 15 kDa. B: the coverage result of the peptide with molecular mass of about 8 kDa.
